# Subakutes Nierenversagen bei einer 40-jährigen nordafrikanischen Patientin

**DOI:** 10.1007/s00108-021-00964-8

**Published:** 2021-02-22

**Authors:** T. Chahoud-Schriefer, T. Wiech, G. Schäfer, S. Harendza

**Affiliations:** 1 III. Medizinische Klinik und Poliklinik, Universitätsklinikum Hamburg-Eppendorf, Martinistraße 52, 20246 Hamburg, Deutschland; 2 Sektion Nephropathologie, Universitätsklinikum Hamburg-Eppendorf, Hamburg, Deutschland; 3 MVZ Infektiologie, Ambulanzzentrum des UKE, Hamburg, Deutschland

**Keywords:** Akutes Nierenversagen, Interstitielle Nephritis, Nierenbiopsie, Urinsediment, Urogenitaltuberkulose, Acute kidney injury, Nephritis, interstitial, Renal biopsy, Urinary sediment, Tuberculosis, urogenital

## Abstract

Eine 40-jährige Patientin aus Eritrea stellte sich zur Abklärung einer unklaren progredienten Niereninsuffizienz vor. Die konservative Diagnostik war nicht wegweisend. Die Nierenbiopsie zeigte eine interstitielle Nephritis, deren Genese sich aufgrund einer leeren Medikamentenanamnese nicht zuordnen ließ. Im Rahmen der Abklärung ergab sich ein Rezidiv der bereits 2015 therapierten Urogenitaltuberkulose. Bei Vorliegen einer interstitiellen Nephritis sollte neben einer Medikamentenanamnese auch an eine Genese im Rahmen von systemischen Infektionen oder Systemerkrankungen gedacht werden.

## Abstract (en)

A 40-year old woman from Eritrea was admitted due to worsening renal function of unknown origin. The basic nephrologic workup provided no further information. Renal biopsy was performed and revealed acute interstitial nephritis (AIN) while no offending medication could be identified. Further investigations showed a recurrence of the urogenitary tuberculosis that had already been in 2015. The finding of AIN in the absence of a typical medical history should raise suspicion of infection-related forms or AIN associated with systemic diseases.

## Anamnese

Eine 40-jährige Patientin wurde uns zur weiteren Abklärung einer progredienten Niereninsuffizienz zugewiesen, die erstmalig vier Monate zuvor dokumentiert worden war. Nach Verlassen des Heimatlands lebte die aus Eritrea stammende Frau seit drei Jahren in einer Flüchtlingsunterkunft. Ein Jahr zuvor hatte eine normale Nierenfunktion mit einem Serumkreatinin von 0,8 mg/dl bestanden. Bei sonstiger Beschwerdefreiheit berichtete die Patientin über chronische diffuse Unterbauchschmerzen, die vor einigen Wochen zu einer Vorstellung in der hiesigen Notaufnahme geführt hatten und als Kolpitis gewertet wurden. Medikamente wurden nicht eingenommen. Nach Aktenlage bestand eine durchgemachte Urogenitaltuberkulose mit Adnexektomie links und Adhäsionen der belassenen Adnexe rechts. Es lag keine B‑Symptomatik vor.

## Untersuchungsbefunde

Die Patientin präsentierte sich afebril in stabilem Allgemeinzustand und leicht adipösem Ernährungszustand. Die Vitalparameter waren unauffällig. Außer leichten diffusen Schmerzen im Unterbauch ergab die *körperliche Untersuchung* keinen wegweisenden Befund. Periphere Ödeme oder eine Lymphadenopathie bestanden nicht.

*Laborchemisch* zeigte sich ein Serumkreatinin von 2,1 mg/dl, das C‑reaktive Protein lag bei 16 mg/l und der Hämoglobinwert bei 11,4 g/dl. Die Albuminurie im *Spontanurin* betrug 2,4 g/g Kreatinin. Das *Urinsediment* zeigte vereinzelt Erythrozyten ohne Akanthozyten oder Zylinder.

**Figure 1:**
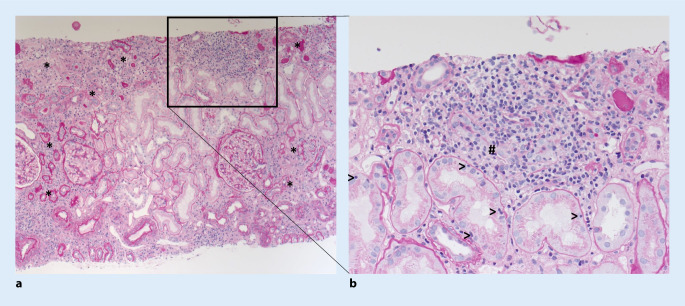

*Abdomensonographisch* waren die Nieren beidseits von normaler Größe, morphologisch bestand kein Hinweis auf eine Harnabflussstörung. Ein Korrelat für die intermittierend geklagten Unterbauchschmerzen fand sich nicht. Bei Vorliegen einer Niereninsuffizienz unklarer Genese wurde die Indikation zur *Nierenbiopsie *gestellt. Diese zeigte eine mittelschwere herdförmige, teils chronisch vernarbende, teils floride nichteitrige interstitielle Nephritis der Rinde (Abb. 1).

## Differenzialdiagnostische Überlegungen

Anhand der laborchemischen und histopathologischen Befunde bestand eine tubulointerstitielle Nephritis unklarer Genese. Eine Medikamentenassoziation konnte bei zuletzt leerer Medikamentenanamnese nicht hergestellt werden. Aufgrund der Vorgeschichte einer Urogenitaltuberkulose wurde eine *Mykobakteriendiagnostik des Urins* veranlasst. Nach einer Latenzzeit von 6 Wochen gelang der kulturelle Nachweis von *Mycobacterium-tuberculosis*-Komplex im Urin. In Zusammenschau der vorliegenden Befunde musste nun von einer interstitiellen Nephritis bei Rezidiv der vorbekannten Urogenitaltuberkulose ausgegangen werden. Die Nierenbiopsie zeigte keine granulomatösen Veränderungen und keine säurefesten Stäbchen.

## Diagnose

-  Tubulointerstitielle Nephritis bei Rezidiv einer Urogenitaltuberkulose mit kulturellem Nachweis von *Mycobacterium-tuberculosis*-Komplex im Urin

## Therapie und Verlauf

Nach infektiologischer Vorstellung wurde eine 4‑fache antituberkulöse Therapie mit Rifampicin, Isoniazid plus Vitamin B_6_, Ethambutol sowie Pyrazinamid, eingeleitet, letztere beiden Substanzen wurden in nierenadaptierter Dosierung gegeben. Eine Human-immunodeficiency-virus-Infektion sowie eine Virushepatitis B und C konnten ausgeschlossen werden. Unter der antituberkulösen Therapie kam es zu Müdigkeit und Emesis, die einer kurzfristigen stationären Behandlung bedurften. Die Therapie wurde nach zwei Monaten auf eine 2‑fach-Therapie mit Rifampicin und Isoniazid/Vitamin B_6_ deeskaliert und schließlich nach 7 Monaten beendet. Unter der kausalen Therapie konnte leider keine Verbesserung der Nierenfunktion erreicht werden, das Serumkreatinin lag zuletzt bei 2,7 mg/dl, die errechnete glomeruläre Filtrationsrate bei 21 ml/min. Eine neuerliche *Mykobakteriendiagnostik* des Urins blieb negativ. Bei fortgeschrittener chronischer Niereninsuffizienz befindet sich die Patientin in engmaschiger nephrologischer Betreuung.

## Diskussion

### Epidemiologie

Die akute tubulointerstitielle Nephritis (ATIN) ist durch das Vorliegen inflammatorischer Infiltrate und eines Gewebeödems im Tubulointerstitium der Niere in Verbindung mit einer akuten Verschlechterung der Nierenfunktion charakterisiert. Es handelt sich um eine häufige und besonders bei älteren Patienten wahrscheinlich deutlich unterdiagnostizierte Entität des akuten Nierenversagens [8]. Die vormals beschriebene klassische klinische Symptomatik eines Hypersensitivitätssyndroms mit Exanthem, Eosinophilie und Fieber ist einem oligosymptomatischen Krankheitsbild bestehend aus unspezifischen Allgemeinsymptomen wie subfebrilen Temperaturen oder leichten Arthralgien bzw. einer gänzlichen Asymptomatik gewichen [11].

Die wahre Inzidenz der ATIN ist schwer zu erheben, da die veröffentlichten Studien größtenteils auf retrospektiven Registerdaten basieren [10, 11]. Hinzu kommt, dass vor allem ältere, gebrechliche und multimorbide Patienten, die bei Polypharmazie häufig ATIN-auslösende Medikamente einnehmen, wie onkologische Patienten unter Chemotherapie [1], meist nicht biopsiert werden. Außerdem variiert die Biopsiehäufigkeit in Abhängigkeit von der Erfahrung und Leitlinie des behandelnden Zentrums deutschland- und weltweit stark [12], sodass oft auf eine Biopsie verzichtet wird.

Die ATIN ist eine häufige und wahrscheinlich deutlich unterdiagnostizierte Entität des akuten Nierenversagens

Die Prävalenz einer histologisch gesicherten ATIN beträgt weltweit 0,5–2,6 % bezogen auf alle Nierenbiopsien [11]. Betrachtet man Biopsien, die bei akutem Nierenversagen durchgeführt wurden, findet sich eine ATIN in 15–27 % der Fälle [10]. Ein Anstieg der Inzidenz ist vor allem in der Altersgruppe der über 65-Jährigen zu verzeichnen, am ehesten bedingt durch eine größere Suszeptibilität der älteren Niere und eine häufig begleitende Polypharmazie [8]. In Europa und Nordamerika dominiert die medikamentös induzierte Form (78 %) gefolgt von der ATIN im Zusammenhang mit einer Systemerkrankung, während in Asien und Afrika infektiöse Ursachen für das Vorliegen einer ATIN die wichtigere Rolle spielen (50 %; [11]).

### Klinische Symptomatik

Die klinische Präsentation der ATIN ist sehr variabel und umfasst neben Verläufen mit fulminantem akutem Nierenversagen und Oligurie auch subklinisch verlaufende und oligosymptomatische Fälle. Lediglich in 5–10 % der Fälle ist die klassische Trias einer Hypersensitivität (Hauterscheinungen, Fieber, Eosinophilie) zu beobachten [9]. In Tab. 1 sind die häufigsten klinischen Befunde im Rahmen der ATIN aufgeführt.

**Table 1:** 

Klinischer Befund	Häufigkeit (%)
Akutes Nierenversagen	100
Akutes Nierenversagen mit Dialysepflichtigkeit	40
Arthralgien	45
Fieber	36
Exanthem	22
Eosinophilie (>500 Eosinophile/mm^3^)	35
Mikrohämaturie	67
Makrohämaturie	5
Leukozyturie	82
Subnephrotische Proteinurie	93

### Ätiologie und Pathogenese

Die Ursachen einer ATIN können in vier Hauptgruppen eingeteilt werden (Tab. 2). Die medikamentös induzierte ATIN repräsentiert mehr als 75 % der Fälle in der westlichen Welt, wobei eine vielfältige und wachsende Zahl auslösender Medikamente beschrieben wurde [12]. Die Symptome treten typischerweise 10–14 Tage nach Exposition auf [12]. Die Latenzzeit ist jedoch variabel und kann zwischen einem Tag nach Antibiotika- und 18 Monaten nach Einnahme nichtsteroidaler Antirheumatika (NSAR) betragen [10]. Der Effekt ist dosisunabhängig und nach Reexposition kommt es zu einer Rekurrenz der ATIN. Prinzipiell kann jedes Medikament eine ATIN induzieren. Die Hauptverursacher sind jedoch Antibiotika und NSAR [3]. Außerdem spielen Protonenpumpeninhibitoren durch ihre weltweite Verbreitung im Rahmen indikationsbasierter Verschreibungen, aber auch durch ihren Einsatz als Over-the-counter-Medikamente eine zunehmende Rolle [11]. Durch die stetig wachsende Zahl neuer onkologischer Medikamente rücken Chemotherapeutika, monoklonale Antikörper und Immuncheckpointinhibitoren ebenfalls zunehmend in den Kreis auslösender Medikamente [1].

**Table 2:** 

*Medikamente (>75* *%)*	
Antibiotika	Penicillin G, Ampicillin, Methicillin, Cephalosporine, Fluorchinolone, Sulfonamide, Rifampicin, Vancomycin, Makrolide
Nichtsteroidale Antirheumatika, Coxibe, Salicylate	Acetylsalicylsäure, Ibuprofen, Naproxen, Mesalazin
Antikonvulsiva	Phenytoin, Carbamazepin
Diuretika	Furosemid, Hydrochlorothiazid, Chlortalidon
Magenschutz, Protonenpumpeninhibitoren	Omeprazol, Pantoprazol, Cimetidin
Chemotherapeutika/Immuntherapie	Carboplatin, Gemcitabin, Methotrexat, Sorafenib, Ipilimumab
Andere	Allopurinol, Aciclovir, Carbimazol
*Infektionen (5–10* *%)*	
Bakterien	Brucellen, *Campylobacter, Escherichia coli*, Legionellen, Mykobakterien, Salmonellen, Streptokokken, Staphylokokken, Toxoplasmen, Yersinien
Viren	Zytomegalievirus, Epstein-Barr-Virus, Hantavirus, „human immunodeficiency virus“, Masern, Polyomavirus
*Systemerkrankungen (10–15* *%)*	Sarkoidose, Sjögren-Syndrom, systemischer Lupus erythematodes
*Idiopathisch (5–10* *%)*	Gegen *Anti-TBM* (tubuläre Basalmembran) gerichtete Erkrankung, *TINU* Syndrom der tubulointerstitiellen Nephritis und Uveitis

Medikamentöser Hauptverursacher der akuten tubulointerstitiellen Nephritis sind Antibiotika und NSAR

Die Pathogenese der Tuberkulose(TB)-assoziierten ATIN ist nicht hinreichend verstanden. Es wird davon ausgegangen, dass indirekte immunologische Mechanismen eine Rolle spielen [4]. Dabei tritt die ATIN häufig spät, das heißt im Rahmen einer fortgeschrittenen TB auf und ist häufig mit einer schlechten renalen Prognose assoziiert [2].

Eine TB ist sehr selten (0,5–1 %) die Ursache einer Niereninsuffizienz [5]. Weitaus häufiger als die Infektionserkrankung selbst ist die medikamentöse tuberkulostatische Therapie Auslöser einer ATIN [7].

### Diagnostik und Differenzialdiagnose

Laborchemisch zeigt sich bei der ATIN ein akutes Nierenversagen unterschiedlichen Schweregrads. In der Urinuntersuchung finden sich typischerweise eine Leukozyturie mit Mikrohämaturie und eine tubuläre Proteinurie (α-Mikroglobulinurie). Mittels Hansel-Färbung (Methylenblau-Eosin) kann eine Eosinophilurie nachgewiesen werden, diese ist allerdings nicht spezifisch für eine ATIN. Der Nachweis von Leukozytenzylindern im Urinsediment ist bei Fehlen von Hinweisen auf eine Pyelonephritis stark verdächtig für das Vorliegen einer ATIN [9].

Sonographisch finden sich normal große bis vergrößerte Nieren mit inhomogen-verdichtetem Parenchym. Eine Diagnosesicherung gelingt letztlich nur mittels Nierenbiopsie. Histopathologisch zeigt sich eine diffuse oder herdförmig akzentuierte tubulointerstitielle Infiltration mit Entzündungszellen (Lymphozyten, Makrophagen, Plasmazellen, Eosinophilen) bei begleitendem tubulointerstitiellem Ödem (Abb. 1). Der Nachweis interstitieller Granulome lässt an eine Sarkoidose oder TB denken, kann in seltenen Fällen aber auch bei medikamentös induzierten Formen vorliegen [10]. Bei der Interpretation der Nierenbiopsie ist wichtig, dass aufgrund eines möglichen „sampling errors“ das Fehlen von Granulomen das Vorliegen einer TB bzw. Sarkoidose keinesfalls ausschließt [12]. TB-assoziierte Fälle einer ATIN ohne Vorliegen von Granulomen wurden bereits beschrieben [4]. Eine ATIN ist eine mögliche Präsentation der Nierentuberkulose, die auch ohne Nachweis von Granulomen beschrieben wurde. In der Literatur sind allerdings nur wenige Fälle dieser Präsentation dokumentiert.

Differenzialdiagnostisch stellt die Abgrenzung zur akuten Tubulusnekrose die größte Herausforderung dar

Differenzialdiagnostisch stellt die Abgrenzung zur akuten Tubulusnekrose die größte Herausforderung dar. Letztere geht nie mit Hypersensitivitätssymptomen einher und das Urinsediment kann zur Unterscheidung hilfreich sein. Fehlt eine Medikamentenexposition, sollten bei ATIN infektiöse Ursachen sowie eine Nierenbeteiligung bei Systemerkrankung (Tab. 2) evaluiert werden und eine entsprechende serologische und mikrobiologische Diagnostik veranlasst werden. Weitere seltenere Differenzialdiagnosen der ATIN sind eine Nierenbeteiligung im Rahmen einer Immunglobulin-G4-assoziierten Erkrankung sowie das Drug-reaction-with-eosinophilia-and-systemic-symptoms(DRESS)-Syndrom [11].

### Therapie und Prognose

Aufgrund der Heterogenität der Erkrankung müssen Therapieempfehlungen sehr differenziert ausgesprochen werden. Randomisierte, kontrollierte Studien fehlen leider gänzlich. Bei der medikamentös induzierten ATIN stellen die Identifikation und das Absetzen des auslösenden Medikaments die Basismaßnahmen dar.

Die Datenlage zum Einsatz von Steroiden stützt sich auf Fallberichte und retrospektive Studien [3, 6]. Verlässliche randomisierte, kontrollierte Studien zum Einsatz von Steroiden fehlen gänzlich, sodass ihr Einsatz kontrovers gesehen wird. Während einige Studien eine raschere und bessere Erholung der Nierenfunktion dokumentieren [3], kann der Erfolg einer Steroidtherapie in anderen Studien nicht bestätigt werden [3]. Auch bei unsicherer Datenlage wird derzeit in den meisten Zentren eine Steroidtherapie begonnen (1 mg/kgKG, Ausschleichen über 4–6 Wochen), sollte sich keine Besserung der Nierenfunktion innerhalb von 3 bis 5 Tagen nach Absetzen des auslösenden Medikaments einstellen. Es gibt Hinweise darauf, dass ein zeitnaher Beginn der Steroidtherapie mit einem besseren renalen Outcome assoziiert ist [6].

Nach Absetzen der auslösenden Medikation kommt es in den meisten Fällen zu einer Erholung der Nierenfunktion. In bis zu 40 % der Fälle wird allerdings nur eine partielle Remission beobachtet, am ehesten bedingt durch eine rasche Transformation der inflammatorischen Läsionen in eine interstitielle Fibrose und Tubulusatrophie, die maßgeblich für die renale Prognose sind [10].

Bei infektiösen Auslösern oder bei Vorliegen einer Systemerkrankung besteht die kausale Therapie in der Behandlung der Grunderkrankung. Insbesondere bei der TB-assoziierten Form der ATIN ist eine rechtzeitige Diagnosestellung für die renale Prognose bedeutsam. Ist eine fortgeschrittene Niereninsuffizienz bei TB-assoziierter ATIN bereits eingetreten (glomeruläre Filtrationsrate < 15 ml/min), werden 75 % der Patienten innerhalb eines Jahres dialysepflichtig [4].

## Fazit für die Praxis

- Die akute tubulointerstitielle Nephritis ist eine häufige und unterdiagnostizierte Entität des akuten Nierenversagens.
- Eine Diagnosesicherung gelingt nur mittels Nierenbiopsie.
- Fehlt eine Medikamentenexposition, sollten infektiöse Ursachen und eine Nierenbeteiligung bei Systemerkrankungen untersucht werden.
- Basismaßnahme ist das Absetzen der auslösenden Medikation oder die Therapie der infektiösen Ursache bzw. zugrunde liegenden Systemerkrankung.
- Eine kurzfristige Steroidtherapie sollte frühzeitig erwogen werden.

## References

[1] Airy M, Raghavan R, Truong LD, Eknoyan G (2013). Tubulointerstitial nephritis and cancer chemotherapy: Update on a neglected clinical entity. Nephrol Dial Transplant.

[2] Chapagain A, Dobbie H, Sheaff M, Yaqoob MM (2011). Presentation, diagnosis, and treatment outcome of tuberculous-mediated tubulointerstitial nephritis. Kidney Int.

[3] Clarkson MR, Giblin L, O’Connell FP (2004). Acute interstitial nephritis: Clinical features and response to corticosteroid therapy. Nephrol Dial Transplant.

[4] Delafosse M, Teuma C, Miailhes P (2018). Severe tubulointerstitial nephritis: tracking tuberculosis even in the absence of renal granuloma. Clin Kidney J.

[5] Eastwood JB, Zaidi M, Maxwell JD (1994). Tuberculosis as primary renal diagnosis in end-stage uremia. J Nephrol.

[6] González E, Gutiérrez E, Galeano C (2008). Early steroid treatment improves the recovery of renal function in patients with drug-induced acute interstitial nephritis. Kidney Int.

[7] Latus J, Amann K, Braun N, Alscher MD, Kimmel M (2012). Tubulointerstitial nephritis in active tuberculosis—a single center experience. Clin Nephrol.

[8] Nasr SH, Muriithi AK, Fidler ME (2014). Clinical characteristics, causes and outcomes of acute interstitial nephritis in the elderly. Kidney Int.

[9] Perazella MA (2014). Diagnosing drug-induced AIN in the hospitalized patient: a challenge for the clinician. Clin Nephrol.

[10] Praga M, Gonzalez E (2014). Acute interstitial nephritis. Core concepts parenchymal kidney dis.

[11] Praga M, Sevillano A, Aunon P, Gonzalez E (2015). Changes in the aetiology, clinical presentation and management of acute interstitial nephritis, an increasingly common cause of acute kidney injury. Nephrol Dial Transplant.

[12] Schmaderer C, Amann K, Heemann U (2015). Akute tubulointerstitielle Nephritis. Nephrologe.

